# Supplementary material for: Experimental warming influences species abundances in a Drosophila host community through direct effects on species performance rather than altered competition and parasitism
Source: PLoS One. 2021 Feb 11;16(2):e0245029. doi: 10.1371/journal.pone.0245029 (PMC7877627; doi:10.1371/journal.pone.0245029)

S1 Fig. Transparent plastic boxes (47cm x 30cm x 27.5cm) with three ventilation holes (15 cm in diameter) covered with insect-proof nylon mesh used as experimental unit allowing parasitoids to attack one of the three experimental vials containing 2-days-old host larvae for 72h

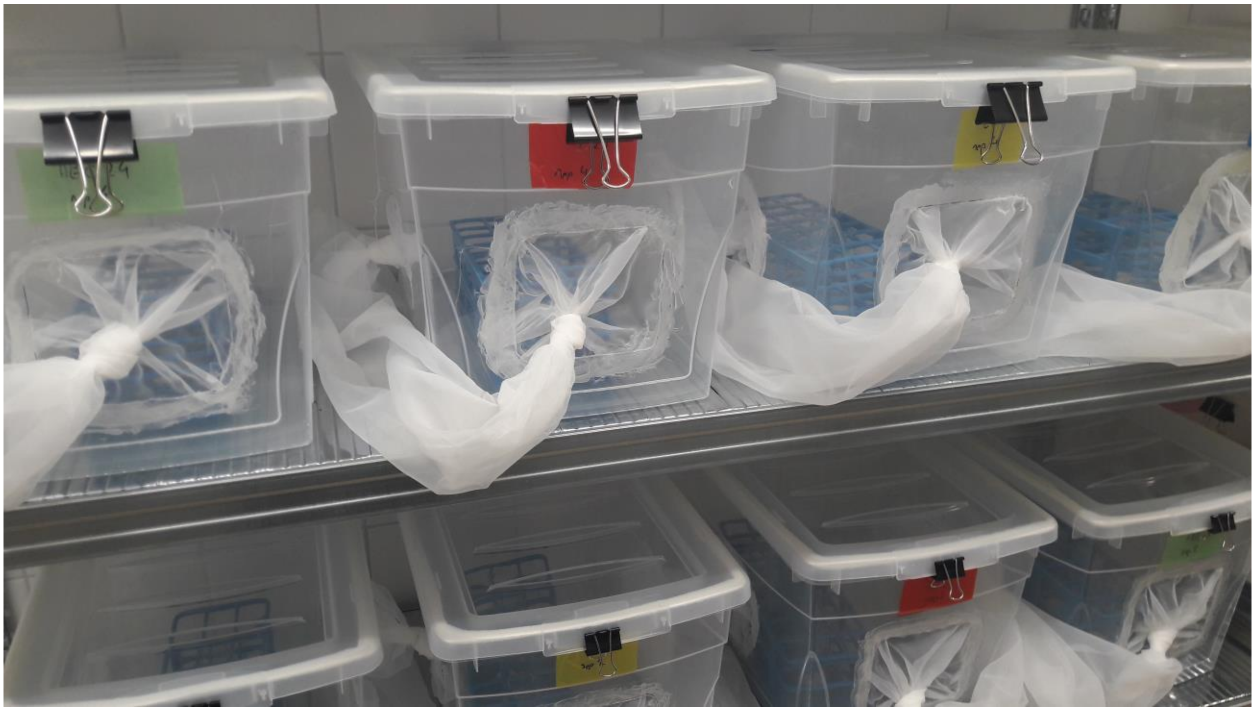

Supplement: S1 Fig — (PDF) [file pone.0245029.s001.pdf]
