# Supplementary material for: Experimental warming influences species abundances in a Drosophila host community through direct effects on species performance rather than altered competition and parasitism
Source: PLoS One. 2021 Feb 11;16(2):e0245029. doi: 10.1371/journal.pone.0245029 (PMC7877627; doi:10.1371/journal.pone.0245029)

S2 Fig. *Drosophila birchii*, *D. pseudoananassae*, and *D. sulfurigaster* pupae photography for morphological identification. Not in scale (photo credit to Jinlin Chen)

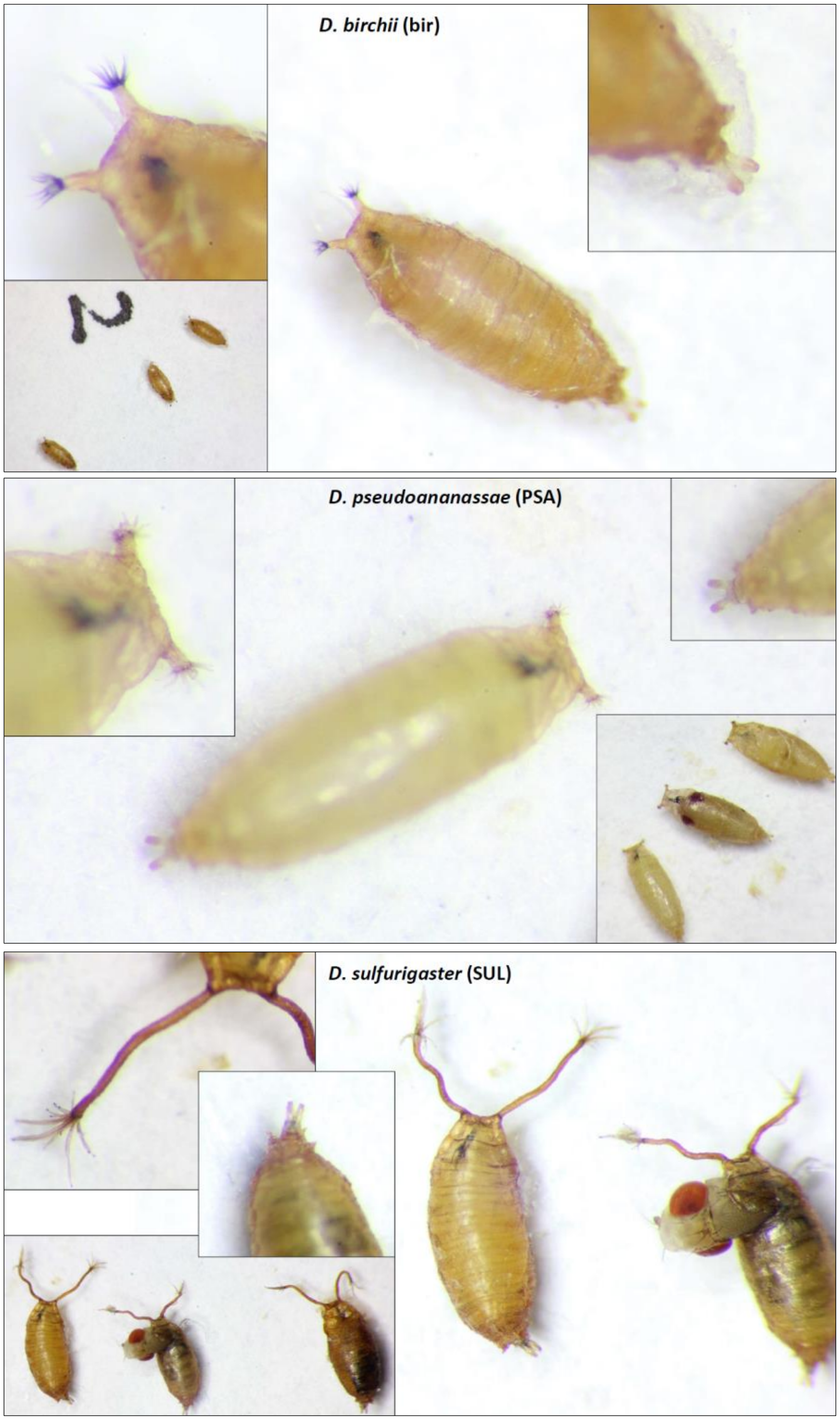

Supplement: S2 Fig — Not in scale (photo credit to Jinlin Chen). (PDF) [file pone.0245029.s002.pdf]
