# Supplementary material for: Experimental warming influences species abundances in a Drosophila host community through direct effects on species performance rather than altered competition and parasitism
Source: PLoS One. 2021 Feb 11;16(2):e0245029. doi: 10.1371/journal.pone.0245029 (PMC7877627; doi:10.1371/journal.pone.0245029)

S3 Fig. Interactive effect of competition with host species, and with presence of parasitoids on mean host body mass. See Figure 1 for detailed description of the treatments. The small points represent the values from each block and each host-parasitoid pair, the large points represent the grand mean, and the bars represent standard errors of the means. Blue: ambient temperature (23°C), red: warming treatment (27°C)

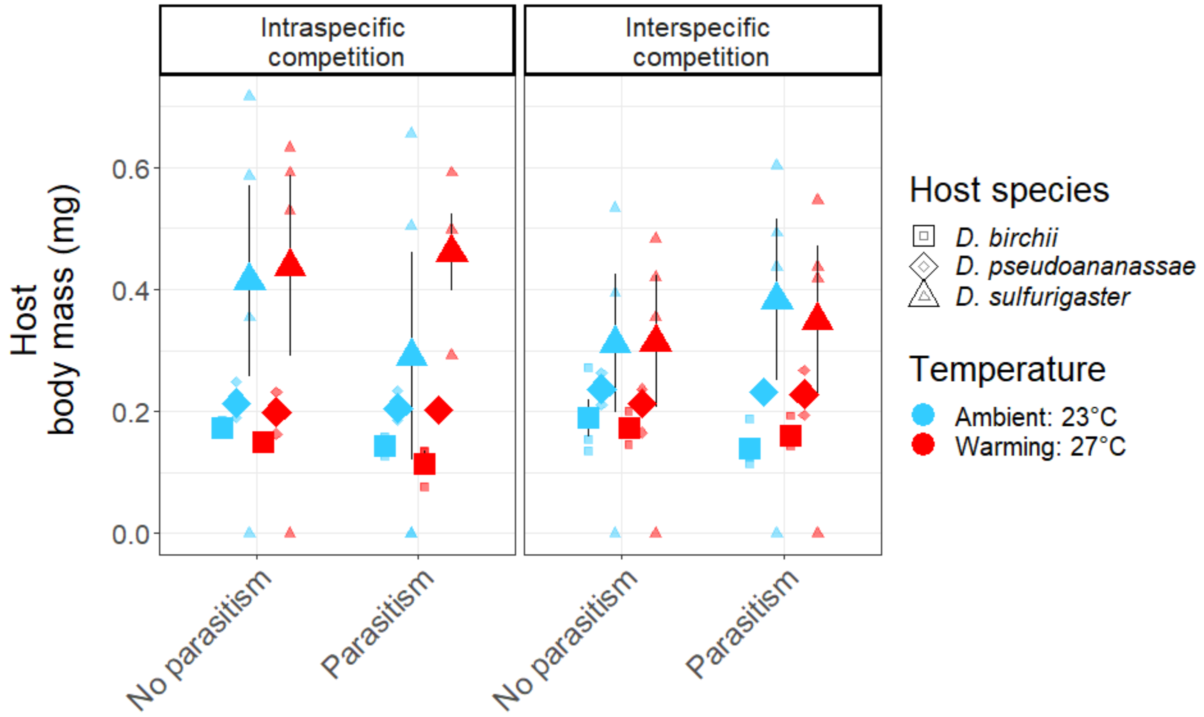

Supplement: S3 Fig — See Fig 1 for detailed description of the treatments. The small points represent the values from each block and each host-parasitoid pair, the large points represent the grand mean, and the bars represent standard errors of the means. Blue: ambient temperature (23°C), red: warming treatment (27°C). (PDF) [file pone.0245029.s003.pdf]
