# Supplementary material for: Experimental warming influences species abundances in a Drosophila host community through direct effects on species performance rather than altered competition and parasitism
Source: PLoS One. 2021 Feb 11;16(2):e0245029. doi: 10.1371/journal.pone.0245029 (PMC7877627; doi:10.1371/journal.pone.0245029)
Supplement: S1 Table — Choice of host number in the main experiment was based on these preliminary data to correspond to strong competition for all host species. (PDF) [file pone.0245029.s004.pdf]

S1 Table. Mean number of offspring per species with 10, 30, 60, 90 or 180 adult hosts (1:1 sex ratio) in a 5 mL host-media glass vial. Choice of host number in the main experiment was based on these preliminary data to correspond to strong competition for all host species.

	**Number of adult hosts**	**Mean number of offspring ± SEM**
D. birchii	10	38 ± 28
	30	45 ± 9
	60	53 ± 19
	90	53 ± 10
	180	40 ± 17
D. pseudoananassae	10	46 ± 27
	30	90 ± 31
	60	126 ± 38
	90	94 ± 33
	180	107 ± 75
D. sulfurigaster	10	40 ± 35
	30	18 ± 11
	60	18 ± 13
	90	35 ± 27
	180	35 ± 19
