# Supplementary material for: Experimental warming influences species abundances in a Drosophila host community through direct effects on species performance rather than altered competition and parasitism
Source: PLoS One. 2021 Feb 11;16(2):e0245029. doi: 10.1371/journal.pone.0245029 (PMC7877627; doi:10.1371/journal.pone.0245029)
Supplement: S2 Table — (PDF) [file pone.0245029.s005.pdf]

S2 Table. Number of observations per temperature, treatments (Intraspecific competition: no interaction between host species; Interspecific competition: direct competition between host species; Parasitism: intraspecific competition with parasitism; All interactions: interspecific competition with parasitism), and host species in the whole dataset, and with the reduced dataset used for analyses (excluding observations with fewer than 10 emerging insects or pupae).

	**Whole dataset**	**Reduced dataset**
**23°C**	48	41
**27°C**	48	42
**Intraspecific competition**	24	21
**Interspecific competition**	24	20
**Parasitism**	24	21
**All interactions**	24	21
D. birchii	32	32
D. pseudoananassae	32	32
D. sulfurigaster	32	19
