# Supplementary material for: Experimental warming influences species abundances in a Drosophila host community through direct effects on species performance rather than altered competition and parasitism
Source: PLoS One. 2021 Feb 11;16(2):e0245029. doi: 10.1371/journal.pone.0245029 (PMC7877627; doi:10.1371/journal.pone.0245029)
Supplement: S3 Table — Degrees or freedom (Df) for each F-ratio are given for each factor and for the error. F values are presented with the significance of the effect: (***) P < 0.001, (**) P < 0.01, (*) P < 0.05, (ns) P > 0.05. (PDF) [file pone.0245029.s006.pdf]

S3 Table. Table showing the effect of temperature (23°C or 27°C), parasitism (presence or absence), competition between host species (intraspecific or interspecific), host species (n = 3), interactions between terms, and block (n = 4), on host abundances, and host frequencies for the whole dataset (without any deleted observations due to *D. sulfurigaster*). Degrees or freedom (Df) for each F-ratio are given for each factor and for the error. F values are presented with the significance of the effect: (***) P < 0.001, (**) P < 0.01, (*) P < 0.05, (ns) P > 0.05.

	Df	Host abundance		Host frequency		Parasitism rate	
**Temperature**	1	0.63	(ns)	0.21	(ns)	5.42	*
**Parasitism**	1	8.50	**	0.00	(ns)	-	-
**Competition**	1	0.11	(ns)	0.00	(ns)	1.25	(ns)
**Host species**	2	27.22	***	97.81	***	2.75	(ns)
**Parasitoid species**	1	-	-	-	-	2.68	(ns)
**Temperature x Parasitism**	1	0.53	(ns)	0.00	(ns)	-	-
**Temperature x Competition**	1	0.10	(ns)	0.00	(ns)	0.04	(ns)
**Temperature x Host species**	2	3.01	(ns)	20.05	***	-	-
**Parasitism x competition**	1	1.61	(ns)	0.00	(ns)	-	-
**Host x parasitoid species**	2	-	-	-	-	22.42	***
**Block**	3	1.02	(ns)	0.00	(ns)	1.66	(ns)
**Df error**		82		82		76	
**R^2**		0.74		0.05		0.10	
