# Supplementary material for: Experimental warming influences species abundances in a Drosophila host community through direct effects on species performance rather than altered competition and parasitism
Source: PLoS One. 2021 Feb 11;16(2):e0245029. doi: 10.1371/journal.pone.0245029 (PMC7877627; doi:10.1371/journal.pone.0245029)
Supplement: S4 Table — (PDF) [file pone.0245029.s007.pdf]

S4 Table. Summary table for mean (± SD) host abundances (Host ab.), individual host body mass (Host BM), total parasitism rate (PR), and parasitism rates of each parasitoid species (*Asobara* sp. and *Leptopilina* sp.) for each temperature (23 and 27°C), treatments (competition: intra or inter, parasitism: present or absent), and host species (*D. birchii, D. pseudoananassae, D. sulfurigaster*).

Temp	Comp	Para	Host sp.	Host ab.	± SD	Host BM	± SD	PR	± SD	PR of *Asb.*	± SD	PR of *Lept.*	± SD
23°C	intra	no	***D. birchii***	114	28	0.174	0.012	-	-	-	-	-	-
			D. pseud.	106	52	0.214	0.025	-	-	-	-	-	-
			D. sulfu.	32	4	0.651	0.092	-	-	-	-	-	-
		yes	***D. birchii***	57	70	0.143	0.022	0.30	0.30	0.29	0.29	0.01	0.01
			D. pseud.	70	69	0.205	0.021	0.30	0.18	0.05	0.09	0.26	0.18
			D. sulfu.	20	9	0.579	0.106	0	0	0	0	0	0
	inter	no	***D. birchii***	109	49	0.190	0.061	-	-	-	-	-	-
			D. pseud.	119	68	0.237	0.023	-	-	-	-	-	-
			D. sulfu.	46	16	0.416	0.108	-	-	-	-	-	-
		yes	***D. birchii***	45	14	0.139	0.034	0.31	0.14	0.17	0.12	0.14	0.12
			D. pseud.	65	64	0.232	0.010	0.26	0.19	0.04	0.04	0.22	0.17
			D. sulfu.	11	6	0.465	0.039	0.28	0.24	0.28	0.24	0.00	0.00
27°C	intra	no	***D. birchii***	69	20	0.151	0.004	-	-	-	-	-	-
			D. pseud.	184	36	0.199	0.030	-	-	-	-	-	-
			D. sulfu.	37	28	0.612	0.029	-	-	-	-	-	-
		yes	***D. birchii***	26	25	0.115	0.034	0.08	0.08	0.06	0.09	0.02	0.04
			D. pseud.	170	13	0.202	0.014	0.11	0.09	0.00	0.01	0.10	0.09
			D. sulfu.	31	31	0.517	0.067	0.28	0.36	0.28	0.36	0.00	0.00
	inter	no	***D. birchii***	74	25	0.173	0.023	-	-	-	-	-	-
			D. pseud.	261	48	0.213	0.032	-	-	-	-	-	-
			D. sulfu.	37	2	0.388	0.046	-	-	-	-	-	-
		yes	***D. birchii***	27	11	0.161	0.022	0.25	0.14	0.16	0.10	0.08	0.06
			D. pseud.	130	33	0.229	0.031	0.16	0.10	0.02	0.02	0.14	0.10
			D. sulfu.	21	11	0.482	0.091	0.16	0.16	0.16	0.16	0.00	0.00
