# Supplementary material for: Experimental warming influences species abundances in a Drosophila host community through direct effects on species performance rather than altered competition and parasitism
Source: PLoS One. 2021 Feb 11;16(2):e0245029. doi: 10.1371/journal.pone.0245029 (PMC7877627; doi:10.1371/journal.pone.0245029)
Supplement: Attachment — Submitted filename: Exp2018_PlosOne_Response to reviewers_v2.docx [file pone.0245029.s009.docx]

Dear Editor,

Thank you for considering our manuscript for publication in *PLOS ONE* and for the opportunity to submit a revised version. We are glad that you consider our responses to the reviewers sufficient. We appreciate the constructive criticisms you have raised regarding our figures and statistical tests, and have made appropriate changes in response as detailed point by point below. We hope that this new revision will allow the manuscript to be published in *PLOS ONE*.

Yours sincerely,

Mélanie Thierry, Nicholas A. Pardikes, Chia-Hua Lue, Owen T. Lewis & Jan Hrček

Additional Editor Comments:

Dear THIERRY, M. and authors

I have gone through the revised manuscript. Comments raised by the reviewers were responded by the authors ok. However, I still have doubts about figures (2, 3 and S7) and statistical tests.

* Thank you for raising those mistakes and oversight. We have corrected the results, figures and the tables as suggested.

Line 227 points out the existence of Tukey's post hoc test, however, I could not find through the text or at the figures any reference to it. Please confirm it and add the information or remove it from the text.

* We corrected the methods, which now reads “Post-hoc multiple comparisons were performed using the *emmeans* package, and P-values were adjusted using the Tukey method.” [lines 227-228 of the new revised manuscript]. We have added the Post Hoc test results throughout the Result section [lines 348-352 of the new revised manuscript].

Figure 2, 3 and S7 labels ascribe (The small points represent the values from each block and each host-parasitoid pair, the large points represent the grand mean, and the bars represent standard errors of the means). However, I could no find a difference between point sizes. Please confirm it and add the information or remove it from the text.

* Apologies, the small points were lost with the format conversion of the figures. We uploaded revised versions of the figures with both point sizes.

Figure 3(b) label legends species as follow (□: Asobara sp. - D. pseudoananassae, ˄: Asobara sp. - D. birchii, +: Asobara sp. - D. sulfurigaster, × Leptopilina sp. - D. pseudoananassae, ◊: Leptopilina sp. - D. birchii, ˅: Leptopilina sp. - D. sulfurigaster). However, figure did not shows it. Please add it to the figure.

* This was the same problem than for the small points raised previously. The revised version of Figure 3 now shows those symbols.

Tables 2 and S5 labels are quite similar (difference consist only "...for the whole dataset". Please explain the difference between them - by means of the whole dataset - in the S5 label so readers could follow the authors' idea easily.

* We have added to the Table S5 label “(without any deleted observations due to *D. sulfurigaster*)”.

Further and regarding to F-values legend [F-values are presented with the significance of the effect: (***) P < 0.001, (**) P < 0.01, (*) P < 0.05, (.) P < 0.1, (ns) P > 0.05"] - I understand that p-values greater than 0.05 are non-significative, so "(.) P < 0.1" does not make sense to me, and I am asking to avoid it, even though R model summary gives it. Then change it at the tables (.) to (ns).

* We changed the P-value significance as suggested.
